# Potential contribution of PrEP uptake by adolescents 15–17 years old to achieving the “Ending the HIV Epidemic” incidence reduction goals in the US South

**DOI:** 10.1371/journal.pone.0288588

**Published:** 2023-11-09

**Authors:** Deven T. Hamilton, Li Yan Wang, Karen W. Hoover, Dawn K. Smith, Kevin P. Delaney, Jingjing Li, Tamika Hoyte, Samuel M. Jenness, Steven M. Goodreau

**Affiliations:** 1 Center for Studies in Demography and Ecology, University of Washington, Seattle, WA, United States of America; 2 Division of Adolescent and School Health, National Center for HIV, Viral Hepatitis, STD, and TB Prevention (NCHHSTP), Centers for Disease Control and Prevention, Atlanta, GA, United States of America; 3 Division of HIV Prevention (DHP), National Center for HIV, Viral Hepatitis, STD, and TB Prevention (NCHHSTP), Centers for Disease Control and Prevention (CDC), Atlanta, GA, United States of America; 4 National Center for HIV, Viral Hepatitis, STD, and TB Prevention (NCHHSTP), Centers for Disease Control and Prevention (CDC), Atlanta, GA, United States of America; 5 Department of Epidemiology, Rollins School of Public Health, Emory University, Atlanta, Georgia, United States of America; 6 Departments of Anthropology and Epidemiology, University of Washington, Seattle, Washington, United States of America; SUNY Downstate Health Sciences University, UNITED STATES

## Abstract

**Background:**

The “Ending the HIV Epidemic” (EHE) initiative seeks to reduce new HIV infections in the U.S. by prioritizing federal resources towards highly impacted populations. Antiretroviral therapy (ART) and pre-exposure prophylaxis (PrEP) are essential for reaching EHE goals. Adolescents are often at increased risk for HIV because they may lack agency in negotiating their sexual partnerships and may not have the same access to treatment and prevention as adults. This study estimates the potential contribution of expanded PrEP coverage among adolescents ages 15–17 to achieving the EHE goals in the South.

**Methods:**

An HIV-transmission model was built to simulate the HIV epidemic in the South. Increased ART and PrEP uptake were systematically varied with and without PrEP eligibility including individuals age<18.

**Results:**

Prioritizing PrEP for adolescents had a negligible impact on incidence. At 50% uptake among eligible adolescents and 90% ART coverage, including adolescents only improved the percentage of infections averted from 80.1% to 80.3%. In 10 of 15 scenarios explored, there was no reduction in new infections when PrEP eligibility was expanded to include adolescents age<18. At 95% ART coverage at the population-level incidence among adolescents declined by over 80%, but PrEP uptake among adolescents did not contribute to additional declines in incidence among adolescents.

**Conclusions:**

Prioritizing PrEP for adolescents did not significantly contribute to reaching EHE incidence reductions goal. Focusing resources to specific adolescent populations at risk, such sexual minority males in high incidence settings, will remain an important public health goal outside the context of EHE.

## Introduction

With antiretroviral medications readily available as an effective treatment for HIV as antiretroviral therapy (ART) and for prevention as treatment as prevention (TasP) or preexposure prophylaxis (PrEP), there is now a push to effectively end the HIV epidemic in the United States. The “Ending the HIV Epidemic” (EHE) initiative seeks to reduce the number of new HIV infections nationally by 75% in 5 years (by 2025) and 90% in 10 years (by 2030) by increasing HIV treatment and prevention resources for the most highly impacted localities and populations [1]. TasP and PrEP are two of the foundations upon which the EHE strategy is built.

Several recent studies have estimated the needed increases in ART coverage in specific sub-populations where HIV incidence is high and the need for additional resources is greatest. A study of men who have sex with men (MSM) in Atlanta [2] concluded that it may be possible to reach the EHE goals with immediate and substantial improvements in HIV screening, PrEP use, and ART care retention but that it will require specifically addressing the HIV service needs of Black MSM [2]. Similarly, a study of city-level HIV transmission for 32 priority metropolitan statistical areas (MSAs) found that improving testing, ART coverage and PrEP coverage among all MSM and persons who inject drugs could reduce incidence by 48% to 90% and that only 13 of the 32 MSAs could achieve greater than 90% reductions in HIV incidence even with large-scale interventions that include heterosexual populations [3]. A study that focused on the South [4], the region of the US with the highest HIV incidence [5], reported that the EHE goals could be achieved in the South with ART coverage above 90% and PrEP coverage > 30%.Like the other studies, they also found that the largest reductions in HIV incidence can be achieved by increasing ART coverage among MSM [4].

One key population of interest not specifically addressed in any of these prior studies is adolescents. In 2017, youth aged 13–24 made up 21% (8,164) of the 38,739 new HIV diagnoses in the United States [6]. In addition, a study of adolescent sexual minority males aged 13–18 years recruited in three major US cities reported an annual HIV incidence density of 3.4/100 person-years [7] and a Chicago area study among MSM ages 16–20 reported HIV incidence was 4.11/100 person years [8]. Adolescents are often at increased risk for HIV because they may lack agency in negotiating their sexual partnerships and may not have the same level of access to treatment and prevention as adults. For example, PrEP was shown to be an effective and safe intervention to prevent HIV [9, 10] but it was almost a decade after PrEP became widely available before the Food and Drug Administration (FDA) approved it for use by adolescents. Several simulation studies have suggested that PrEP use by adolescent sexual minority males (ASMM) could reduce a significant number of HIV infections in this population and that PrEP use could also contribute to reducing racial disparities [11–13].

It has been argued that there is a moral imperative to make efficacious HIV prevention methods like PrEP accessible to all persons with PrEP indications, including adolescents so that they are protected during periods of high risk of HIV acquisition [14–16]. Two recent review articles have also made the case that PrEP should be part of combination prevention packages for adolescents [17] and that it should be a routine topic of discussion between health care providers and their adolescent patients [18]. However, it has not been demonstrated that including adolescents as a priority population in the context of EHE will significantly contribute to reductions in HIV incidence.

To answer this question, we conducted an agent-based simulation study. In a prior study seeking greater clarity on how expanded ART and PrEP might impact race and sex disparities, we used a simulation to predict race- and sex-specific HIV trajectories given increased ART and PrEP use by different race and sex groups in the South [4] which currently experiences the greatest burden of HIV of any U.S. region, and lags behind in providing quality HIV prevention services and care [5, 19]. Building on that model, in this study we evaluate the potential contribution of PrEP uptake by adolescents younger than 18 years of age to achieving the EHE incidence reduction goals.

## Methods

We developed a stochastic, agent-based network model to simulate the HIV epidemic in the South. The model was built on the EpiModel [20] and *statnet* [21, 22] software packages. The EpiModelHIV R package was used to incorporate the relevant HIV-specific epidemiology for this study: (github.com/EpiModel/EpiModelHIV). Similar models have been used previously to model HIV and STI interventions and outcomes in other contexts [2, 13, 23]. Full details of the model, data sources, and parameters are available elsewhere [4] but we provide a brief general overview here, including adaptations made to the model for this work.

The simulation included 500,000 individuals with the same age, sex, and race composition as the 15–65-year-old population in the South reported by the US census. The population was 50.6% women, 45.9% heterosexual men and 3.6% MSM [24] and men who have sex with men and women (MSMW) [25]. Women who have sex with women were not included in the simulation given their low HIV burden. Adolescents between 15 and 18 made up 8.3% of the male population and 7.8% of the female population. Sexual network and behavioral parameters were estimated from the National Survey of Family Growth (NSFG) (2011–2017) [26] and ARTnet [27] surveys for heterosexuals and MSM respectively. NSFG respondents ≥18 years provide informed consent; respondents ages 15–17 provide assent after parental permission. NSFG procedures were approved by the National Center for Health Statistics Research Ethics Review Board. ARTnet is an anonymous cross-sectional web-based survey of HIV-related risk behaviors, testing, and use of prevention services among men who have sex with men (MSM) in the United States. The ARTnet survey was conducted from 2017 to 2019 and recruited MSM who have completed the American Men’s Internet Survey (AMIS) study. The Emory University Institutional Review Board approved the study. Our simulation study informed by de-identified, publicly-available data is not considered human subjects research.

The model included six networks and nine different partnership types (e.g., main partnerships, casual (but persistent) partnerships, and one-time sexual contacts between heterosexuals, MSM, and MSMW and heterosexual females). Formations of new partnerships were conditional on current partnerships, and each partnership had type-specific within-partnership behaviors (e.g., HIV status disclosure, condom use, coital frequency) and partnership duration. The networks for main and casual partnerships were modeled using temporal exponential random graph models (TERGMs) [28] while the one-time partnerships were modeled with exponential random graph models (ERGMs) [21, 22]. Each network model was estimated directly from the egocentric data provided by the NSFG and ARTnet surveys. Both the TERGM and ERGM models and the estimation process are described in detail in previous publications [4].

The formation of partnerships, specifically by adolescents, were governed by four terms in each of the network models. First, a factor for the overall propensity of individuals in each of six age categories (15–18, 19–24, 25–34, 35–44, 45–54, and 55–64) to form partnerships. Adolescents were far less likely to form either main or one-time partnerships compared to other older age categories. Second, a factor for the propensity of individuals at age 15 to form partnerships. This factor was used as a tuning parameter to increase the log odds of forming partnerships by new individuals entering the model at age 15. The additional parameter was needed to account for relationships that would have started prior to age 15 if younger ages were represented in the model. Third, a set of mixing terms indicating the propensity of individuals in each age group to form partnerships with individuals in the same age group. More than half of partnerships that included an adolescent were with other adolescents. Finally, a term for the average difference in age in a partnership (offset on networks of heterosexual partnerships to capture age asymmetry in heterosexual partnerships).

Demographic change included entry at age 15, aging, and exit due to mortality or aging beyond the 15–65 age range. Intrahost epidemiology included the natural progression of disease within persons with HIV (PWH) in the absence of clinical intervention. The main component of disease progression explicitly modeled for this study was HIV viral load, which controlled both interhost epidemiology (HIV transmission rates) and disease progression. The clinical epidemiological processes in the model included testing/diagnosis, linkage to care, treatment/PrEP initiation, adherence, and HIV viral suppression.

We simulated increasing ART coverage and PrEP use with two approaches and tested each approach with PrEP restricted to adults and with PrEP available to adolescents ≥ age 15. The first approach was to increase both ART coverage and PrEP use simultaneously; we increased ART coverage from 60% to 100% uniformly across all demographic groups and simultaneously increased PrEP coverage from 10% to 50%. In the second approach we increased ART to 90% and 95% coverage and increased PrEP use incrementally from 10% to 50% with both levels of ART coverage. Table 1 provides an overview of all of the simulated scenarios. The outcomes of interest were the total number of new HIV infections over 8 years of simulation (i.e., new HIV infections by 2030), the percentage of infections averted (PIA), the number of infections averted per 100K person years at risk (NIA), and the annual HIV incidence rates at the end of 8 years of simulation (at year 2030). All of the outcomes are reported as means over 50 simulations and include 80% simulation intervals (SI) which indicate the range within which 80% of the simulated results fall. The SI are not a formal statistical indication of uncertainty; rather they indicate the range for each outcome given the level of stochastic variability in the model.

**Table 1:** Analyses exploring the potential impact of changes in antiretroviral therapy (ART) and pre-exposure prophylaxis (PrEP) coverage on achieving the “Ending the HIV Epidemic” goals.

Scenario^1^	ART coverage	PrEP coverage
1	60%	10%
2	70%	20%
3	80%	30%
4	90%	40%
5	100%	50%
6	90%	10%
7		20%
8		30%
9^2^		40%
10		50%
11	95%	10%
12		20%
13		30%
14		40%
15		50%

1 Each scenario was run with PrEP restricted to indicated adults age 18+ and with PrEP available to indicated persons age 15+.

2 Scenario 9 is the same as scenario 4. It is included separately to facilitate comparisons.

In the baseline simulation mean ART coverage among those infected was 63.6%, 67.9% and 67.9% among Non-Hispanic Black (NHB), Latino/Hispanic and White/Other MSM respectively, 50.0%, 20.6%, and 29.6% among NHB, Latino/Hispanic and White/Other heterosexual males respectively, and 41.4%, 27.1%, and 21.8% among NHB, Latina/Hispanic and White/Other heterosexual females respectively. These coverage rates were outcomes generated by the simulation after the model was calibrated to empirical target statistics for the continuum of care including demographic-group specific testing, linkage to care, and retention rates, and reported overall ART coverage [4, 29–31]. Following ART initiation in the baseline simulation individuals on ART are assigned different levels of ART adherence based on empirical estimates [4]. In each counterfactual scenario with fixed ART coverage the proportion of PWH in the intervention-eligible population on ART is calculated at each time step. If the proportion is less than the fixed coverage, PWH in the intervention eligible population are selected at random to initiate ART. Individuals initiating ART as part of the hypothetical intervention are all assigned the highest level of adherence under the assumption that whatever intervention is used to achieve this new level of ART coverage under EHE will also increase adherence. Throughout the simulation the remainder of the continuum of care continues to operate as it does under the baseline conditions with individuals testing, beginning treatment, engaging in care and discontinuing care. Individuals initiating ART through the continuum of care rather than as a result of the counterfactual intervention that sets a fixed level of coverage adhere to ART based on empirically derived estimates of adherence. If, through this background continuum of care, the proportion of individuals on ART exceeds the fixed coverage, individuals on ART are randomly selected to discontinue ART. The 60% ART coverage rate was selected as our lower bound because it is the closest round value to current MSM coverage rates and thereby reflects an approximation for getting the heterosexual population to ART coverage similar to current coverage among MSM. It is worth noting that this 60% thus reflected a slight decrease in ART coverage among MSM.

The baseline level of PrEP coverage was based on findings from Siegler el al. [32] who reported a rate of 21 PrEP users per 100,000 individuals in the South in Q4 of 2017 overall and a rate of 1.9 per 100,000 females. We assumed that the rate of PrEP use among heterosexual males more closely resembles that of females than MSM and used the 1.9/100,000 rate for heterosexual males as well. After determining the expected number of heterosexual males and females on PrEP using these rates, we subtracted the heterosexuals from the total number of reported PrEP users and used the remainder to determine the rate for MSM (1351.2 per 100,000). To account for differences in PrEP use by race we used the proportions of indicated individuals currently using PrEP reported in the CDC’s HIV Surveillance Supplemental report [33] (5.9% Black, 10.9% Hispanic, 42.1% White/Other) and applied the relative proportions to the three different race groups for the MSM, heterosexual males and heterosexual females. MSM in the simulation were eligible for PrEP if they were HIV-negative, age > 18, and either in a monogamous partnership with a partner who had not tested in the past 6 months, or they were in 2 or more concurrent partnerships. Heterosexuals in the simulation were eligible if they were HIV-negative, age > 18 and either in a casual partnership with a partner who had not tested in 6 months, or they were in 2 or more concurrent partnerships. Eligibility for both heterosexuals and MSM was then expanded to include individuals age ≥ 15 that also met the other inclusion criteria.

In scenarios with fixed levels of PrEP, coverage was calculated using the PrEP-eligible population as the denominator. At each timestep in the simulation PrEP eligibility was evaluated for all individuals not currently eligible for PrEP. For those already determined to be eligible, eligibility was reassessed annually. If coverage was below the fixed coverage level, eligible individuals were randomly selected to initiate PrEP. PrEP discontinuation occurred both stochastically and with a change in eligibility.

### Ethics statement

This research does not involve human subjects and did not require an Institutional Review Board approval or a determination of exempt status by the University of Washington.

## Results

In our baseline scenario, the total number of new HIV infections was 549.7 (80%SI: 487.8, 600.8). The first set of scenarios increased both ART and PrEP simultaneously. This resulted in a largely linear decline in the number of new infections from baseline to 517.6 (80%SI: 462.6, 566.7) at 60%/10% ART/PrEP coverage and to 31.3 (80%SI: 23.8, 40.3) at 100%/50% ART/PrEP coverage. When PrEP eligibility was expanded to include adolescents ages ≥15 the linear decline in the number of new infections was almost identical with 521.2 (80%SI: 469, 583.2) new infections at 60%/10% ART/PrEP coverage and 30.8 (80%SI: 21.6, 39.3) at 100%/50% ART/PrEP coverage. The PIA was 94.3% (80%SI: 92.4, 95.6) at 100%/50% ART/PrEP without PrEP use by adolescents compared to 94.4% (80%SI: 92.5, 95.9) when adolescents where eligible for PrEP (Table 2). The average number of new infections over 50 simulations under each of the 5 ART/PrEP combinations where not demonstrably different irrespective of whether PrEP eligibility started at age 15 or at age 18. In fact, of the five scenarios, there were more new infections on average in four when PrEP was available at age 15. This does not indicate that earlier access to PrEP increases incidence, but rather that any true differences in incidence are far smaller than the random variation in the model.

**Table 2:** Reductions in new HIV infections associated with universal increases to viral suppression through increased antiretroviral therapy (ART) coverage and HIV acquisition reduction through pre-exposure prophylaxis (PrEP) uptake by adults and adults and adolescents in combination.

ART coverage (%)	PrEP coverage (%)^1^	Total number of new HIV infections (mean & 80% simulation intervals)^2^	Percent of infections averted (mean & 80% simulation intervals)	Number of infections averted / 100K (mean & 80% simulation intervals)	Total number of new HIV infections (mean & 80% simulation intervals) ^2^	Percent of infections averted (mean & 80% simulation intervals)	Number of infections averted / 100K (mean & 80% simulation intervals)
**PrEP eligibility**		**Adults 18 + year old**			**Adults and adolescents 15 + year old**		
**Baseline**	**Baseline**	549.7 (487.8, 600.8)	NA	NA	549.7 (487.8, 600.8)	NA	NA
**60**	**10**	517.6 (462.6, 566.7)	5.5 (-10.2, 18.8)	0.8 (-1.3, 2.8)	**521.2 (469, 583.2)**	4.9 (-9.2, 16.4)	0.7 (-1.2, 2.4)
**70**	**20**	353.4 (302, 406.7)	35.4 (22.7, 47.8)	4.9 (3, 7)	**354.6 (319.6, 391.8)**	35.2 (24, 43)	4.9 (3.1, 6.3)
**80**	**30**	221.1 (187.8, 264.8)	59.6 (48.7, 67)	8.2 (6.1, 9.8)	**230 (206.4, 258.7)**	58 (50.2, 64)	8 (6.3, 9.5)
**90**	**40**	116.9 (99, 140.3)	78.6 (73.2, 82.5)	10.9 (9.2, 12.3)	**119.2 (98.2, 143.2)**	78.3 (72.9, 81.8)	10.8 (9.2, 12)
**100**	**50**	**31.3 (23.8, 40.3)**	94.3 (92.4, 95.6)	13 (11.5, 14.3)	30.8 (21.6, 39.3)	94.4 (92.5, 95.9)	13 (11.5, 14.3)
**90**	**10**	**159.9 (123.6, 192.5)**	70.8 (64.6, 77)	9.8 (8.2, 11.3)	156.9 (127.8, 188.8)	71.4 (64.3, 76.3)	9.9 (8.1, 11.1)
	**20**	144.5 (121.6, 166.8)	73.6 (68.2, 78.2)	10.2 (8.5, 11.6)	**148.8 (128.6, 172.2)**	72.8 (66.1, 77.2)	10.1 (8.3, 11.4)
	**30**	126.1 (100, 155.5)	77 (70.5, 81.8)	10.6 (9, 12.1)	**130.3 (106.6, 155.5)**	76.2 (70.4, 80.6)	10.5 (8.9, 11.9)
	**40**	116.9 (99, 140.3)	78.6 (73.2, 82.5)	10.9 (9.2, 12.3)	**119.2 (98.2, 143.2)**	78.3 (72.9, 81.8)	10.8 (9.2, 12)
	**50**	**108.8 (86.6, 133.2)**	80.1 (74.2, 84.7)	11.1 (9.3, 12.6)	107.8 (84.2, 132.2)	80.3 (74.5, 85)	11.1 (9.2, 12.7)
**95**	**10**	99.4 (79.6, 124.7)	81.9 (76.8, 85.5)	11.3 (9.8, 12.6)	**103.4 (79.4, 127.3)**	81.1 (76.1, 85.3)	11.2 (9.5, 12.6)
	**20**	**91.4 (73.4, 110.8)**	83.3 (79.3, 87)	11.5 (9.9, 12.9)	90.3 (72.8, 111.3)	83.5 (79.9, 86.6)	11.5 (9.9, 12.7)
	**30**	81.2 (62.6, 106.5)	85.2 (79.8, 88.6)	11.8 (10.1, 13.1)	**83.8 (67.4, 103.7)**	84.7 (80, 87.9)	11.7 (10.1, 13.1)
	**40**	73 (56.6, 90.5)	86.7 (83.2, 90)	12 (10.5, 13.4)	**74.3 (57.4, 90.8)**	86.4 (83.3, 89.8)	11.9 (10.3, 13.3)
	**50**	**67.6 (53.4, 83.7)**	87.6 (84.3, 90.4)	12.1 (10.5, 13.5)	67.2 (53.2, 81.5)	87.7 (84.5, 90.3)	12.1 (10.6, 13.5)

^1^Prep coverage is calculated using the number of individuals eligible for PrEP as the denominator.

^**2**^The total number of new infections in bold indicate when the number is greater when comparing the same PrEP and ART coverage with and without PrEP use among adolescents ages 15–17.

When ART was increased to 90% and PrEP was incrementally raised from 10% to 50%, we found similar patterns to the previous analysis in that new infections declined in a roughly linear fashion. The vast majority of the reduction was gained with improved ART coverage. At 90%/10% ART/PrEP coverage new infections declined by 70.8% (80%SI:64.6, 77) but increasing PrEP to 50% coverage only improved the PIA to 80.1% (80%SI: 74.2, 84.7). There was no clear reduction in new infections when PrEP eligibility was expanded to include adolescents < age 18. Similar to the above, in three of five scenarios there were more new infections on average with expanded eligibility for adolescents simply by chance.

**Fig 1:**
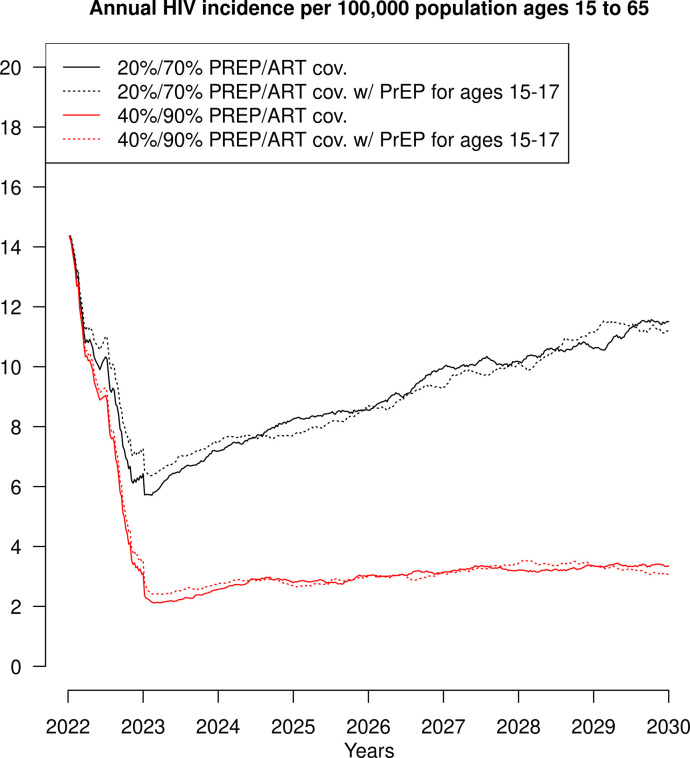
The annual HIV incidence per 100,000 population ages 15 to 65 when antiretroviral therapy coverage is 70% and pre-exposure prophylaxis coverage is 20% with and without adolescents ages 15 through 17 included in the uptake of pre-exposure prophylaxis and the annual HIV incidence per 100,000 population ages 15 to 65 when antiretroviral therapy coverage is 90% and pre-exposure prophylaxis coverage is 40% with and without adolescents ages 15 through 17 included in the uptake of pre-exposure prophylaxis.

Fig 1 shows the average annual HIV incidence among the entire population in two of the scenarios tested, one in which ART coverage was 70% and PrEP coverage was 20% and a second in which ART coverage was 90% and PrEP coverage was 40%. The scenarios are indicated by the color of the line, the solid lines indicate scenarios where adolescents less than age 18 did not receive PrEP, and the dashed lines indicate scenarios where adolescents less than age 18 did receive PrEP. In both coverage scenarios the incidence lines indicating whether or not adolescents received PrEP or not are essentially the same with the exception of stochastic variation.

The annual incidence/100K adolescents age 15–17 during the last year of simulation and the change in incidence in each scenario are shown in Table 3. In our baseline model, annual incidence was 9.7/100K adolescents (80%SI: 2.2, 22.7). The wide range of the 80% SI indicates large variations in outcomes among adolescents due to stochasticity in the simulation. At 60%/10% ART/PrEP coverage incidence declined by 5.6% when PrEP was only available to adults but 13.8% when PrEP was available at age 15. Across all scenarios this was the largest difference in the percent reduction in incidence when adolescents were eligible for PrEP compared to when they were not. However, there was a persistent trend. In 12 of the 15 scenarios the incidence among adolescents declined to a greater degree when they were eligible for PrEP, although the difference was generally just a few percent. For example, with 95%/20% ART/PrEP coverage incidence declined 86.6% among adolescents when they were not eligible for PrEP and 90.3% when they were.

**Table 3:** Reductions in the HIV incidence rate among adolescents ages 15–17 years associated with universal increases to viral suppression through increased antiretroviral therapy (ART) coverage and HIV acquisition reduction through pre-exposure prophylaxis (PrEP) uptake among adults and among adults and adolescents in combination.

Intervention		PrEP 18+ year old		PrEP 15+ years old	
ART coverage (%)	PrEP coverage (%)	Annual incidence per 100K adolescents during the final year of simulation (mean and 80% simulation intervals)^1^	Reduction in incidence	Annual incidence per 100K adolescents during the final year of simulation (mean and 80% simulation intervals) ^1^	Reduction in incidence
**Baseline**	**Baseline**	9.7 (2.2, 22.7)	NA	9.7 (2.2, 22.7)	NA
**60**	**10**	**9.1 (1.5, 17.6)**	5.56%	8.3 (1.3, 19.5)	13.77%
**70**	**20**	6.9 (0.6, 15.8)	28.48%	**7.7 (0.1, 24.4)**	20.34%
**80**	**30**	**4.5 (0, 14.1)**	53.56%	3.8 (0, 10.1)	60.60%
**90**	**40**	**2.4 (0, 8.6)**	75.59%	1.5 (0, 5.1)	84.01%
**100**	**50**	**0.1 (0, 0.6)**	99.25%	0 (0, 0.3)	99.64%
**90**	**10**	2.4 (0, 8.4)	75.12%	**2.8 (0, 9.8)**	70.99%
	**20**	**3.1 (0, 9.5)**	68.38%	1.4 (0, 6.1)	85.47%
	**30**	1.8 (0, 5.9)	81.52%	2.2 (0, 7.3)	77.65%
	**40**	**2.4 (0, 8.6)**	75.59%	1.5 (0, 5.1)	84.01%
	**50**	**1.7 (0, 5.9)**	82.14%	1.2 (0, 5.9)	87.18%
**95**	**10**	**1.6 (0, 6.8)**	83.23%	1.4 (0, 5.9)	85.66%
	**20**	**1.3 (0, 5)**	86.59%	0.9 (0, 4.1)	90.27%
	**30**	**1.6 (0, 6.8)**	83.35%	0.6 (0, 2.7)	94.10%
	**40**	**1.2 (0, 5.5)**	87.41%	1 (0, 5.3)	90.17%
	**50**	**1.4 (0, 6)**	85.70%	1 (0, 3.5)	89.91%

^**1**^Annual incidence per 100K adolescents ages 15–17 in bold indicate when the incidence rate is greater when comparing the same PrEP and ART coverage with and without PrEP use among adolescents age 15–17.

## Discussion

Our analyses indicate that it is possible to reach the EHE goal of a 75% reduction in new HIV infections among 15-65-year-olds in the South by increasing ART coverage to 90% and PrEP coverage to 30%, or ART coverage to 95% if there is less than 10% PrEP coverage. Incidence among adolescents also declines by more than 75% even if adolescents are not eligible for PrEP. Expanding PrEP eligibility to include adolescents had little observable impact on the HIV epidemic and there were no ART/PrEP coverage scenarios in which EHE goals were achieved only when adolescents had access to PrEP. The eligibility criteria for PrEP in our simulation were very broad, which resulted in 17% of all 15-17-year-old adolescents being on PrEP when coverage was set to 50%, and even at that level of PrEP use the impact was negligible. Such widespread PrEP use would also be extremely expensive, and based on our finding, contribute little to achieving the EHE goals.

Despite our findings that PrEP use by adolescents will not significantly impact the HIV epidemic in the South, for those adolescent populations that are at increased risk for acquiring HIV, like ASMM, PrEP may be an invaluable HIV prevention tool. In 2017, 81% of youth diagnosed with HIV were classified as in the male-to-male sexual contact risk category and simulations have suggested that PrEP use by 16-18-year-old ASMM could have prevented 35.1% of infections among ASMM over ten years in high incidence settings [11]. Recent estimates also suggest that MSM make up just 3.57% of the male population in the South [24] so a more focused approach prioritizing those most at risk may be more practical. The National HIV Behavioral Surveillance (NHBS) study also estimated that HIV prevalence among 18–24-year-old Black and White MSM as 26% and 3%, respectively [34] so directing additional prevention resources towards ASMM, and particularly Black ASMM, could reduce HIV infections among adolescents and reduce racial disparities [12].

However, even a more narrowly focused approach to PrEP for adolescents may face challenges [35]. Recruitment of adolescents to enroll in a PrEP program can be difficult, thereby limiting uptake [36] and developing non-stigmatizing messaging focused on adolescents most at risk may be difficult without additional research. Health care providers are also at times hesitant to prescribe medication to prevent HIV infection, due to concerns about side effects [9, 37]. In addition, the physicians who are best trained and most willing to prescribe PrEP tend to be HIV specialists, while those who regularly care for HIV-negative patients (e.g., primary care, general practitioners and pediatricians) are often not trained to provide PrEP [38]. Most adolescents also have infrequent interactions with healthcare providers and have never had an HIV test [39]. Adherence may also be challenging for adolescents. The ATN 113 safety and feasibility study that evaluated the use of TDF/FTC by US adolescents aged 15–17 years found that only 41.6% of participants were highly adherent (≥4 doses/week) [40]. High adherence to oral TDF/FTC is essential for efficacy and suboptimal adherence may result in HIV acquisition or potentially drug resistance.

Despite the challenges to providing PrEP to adolescents, the US Preventive Services Task Force released an “A” rating for PrEP for adolescents who are at risk for HIV [41, 42]. Current CDC guidelines recommend that all patients be informed of PrEP and that PrEP is indicated for anyone who has had anal or vaginal sex in past 6 months and any of the following: 1) An HIV-positive sexual partner (especially if partner has an unknown or detectable viral load), 2) A bacterial STI in past 6 months 3) A history of inconsistent or no condom use with sexual partner(s); has shared injection equipment; or for anyone who requests it [41]. However, given the negligible contribution generalized PrEP programs for adolescents would have on reaching the EHE targets and the barriers such programs are likely to face, the EHE would be best served by directing resources towards adults at risk for HIV and potentially a narrowly focused subset of adolescents. Prior studies have indicated that PrEP can reduce HIV incidence among ASMM, and to a greater extent Black ASMM, because of higher HIV incidence [12] and, PrEP use in this population has also been shown to have favorable cost-effectiveness ratios in communities with high HIV burden [43]. This suggests that in specific contexts, outside of the national incidence reduction goals set by the EHE initiative, PrEP programs prioritizing some adolescent populations may reduce incidence within those priority populations and contribute to reducing disparities.

This study had several limitations. Our model did not include people who inject drugs or trans-identifying individuals as important priority populations. The very recent increase in trans and non-binary identifying adolescents makes this intersection particularly salient for future research in this area [44]. Other STI were also not included in this model which can both increase the rate of HIV transmission and serve as a risk indicator for screening and PrEP [45]. This model did not include social determinants of health which can significantly contribute to additional heterogeneity in risk in the population at large and among adolescents. PEP was also not a prevention option in this model. There is limited data on the performance of PEP based on timing and adherence, so modeling the prevention profile will be difficult in the near term. However, PEP could be an important contribution to reducing new infections and it has several unique advantages including a short adherence window, it does not require planning, and it does not require foreknowledge of a potential exposure. Lastly, contrary to recent trends which have shown decreasing numbers of HIV diagnoses in 2009–18 [5] and projections in other modeling studies [3] which have predicted declining incidence over the next decade, our reference scenario projected an increase over the next 8 years, all else being equal. Our divergent findings are likely a result of our calibration method which calibrated the model to current prevalence and incidence assuming that current reported behavior and the continuum of care were in place from the beginning of the epidemic rather than co-evolving with the epidemic. Given these additional barriers to transmission at the outset our model may overestimate transmission and consequently underestimate the impact of additional viral suppression and PrEP use.

Focusing significant resources on enrolling adolescents in PrEP programs is not likely to contribute to the reduction in incidence that is the goal of the EHE. However, in 2019 44.3% of adolescent with HIV were not aware of their infection status [19] and data from the 2019 Youth Risk Behavior Survey indicate that only 9.4% of high school students had ever tested for HIV, only 8.6% had been tested for an STI in the prior year, and 46% of sexually active high school students did not use a condom at last sexual intercourse [46]. There is clearly a need to improve sexual and reproductive health outcomes for adolescents. Focusing on health care provider training and resources to initiate more comprehensive discussions of sexual health may improve multiple dimensions of health outcomes among adolescents including HIV and STI testing, linkage to care for the 44.3% of undiagnosed adolescent, in addition to improved outcomes for reproductive and sexual health. A more general approach to improving adolescent sexual health including HIV testing also provides a natural segue into discussions of PrEP for those at risk who could most benefit from it.
